# Acute hip fracture surgery anaesthetic technique and 30-day mortality in Sweden 2016 and 2017: A retrospective register study

**DOI:** 10.12688/f1000research.15363.2

**Published:** 2018-08-15

**Authors:** Caroline Gremillet, Jan G. Jakobsson

**Affiliations:** 1 Department of Anaesthesia & Intensive Care, Institution for Clinical Sciences, Danderyds University Hospital, Karolinska Institutet, Stockholm, 18288, Sweden

**Keywords:** acute hip fracture, anaesthetic technique, neuraxial anaesthesia; spinal, epidural, general anaesthesia, 30-day mortality

## Abstract

**Background:** Hip fractures affect 1.6 million patients yearly worldwide, often elderly with complex comorbidity. Mortality following surgery for acute hip fracture is high and multifactorial; high age, comorbidities and complication/deterioration in health following surgery. Whether the anaesthesia technique affects the 30-day mortality rate has been studied widely without reaching a consensus. The primary aim of this study was to determine anaesthetic techniques used in Sweden and their impact on the 30-day mortality rate in elderly undergoing acute hip fracture surgery. Other aims were to study the impact of age, gender, ASA class, fracture type and delay in surgery on the 30-day mortality rate.

**Methods:** Data from 13,649 patients ≥50 years old who had undergone acute hip fracture surgery and been reported to Swedish perioperative register (SPOR) between 2016 and 2017 were analysed.

**Results:** The most commonly used anaesthetic technique was neuraxial anaesthesia (NA; 11,257, 82%), followed by general anaesthesia (GA; 2,190, 16%) and combined general and neuraxial anaesthesia (CA; 202, 1.5%) out of the 13,649 studied. The 30-day mortality rate was 7.7% for the entire cohort; GA 7.8%, NA 7.7% and CA 7.4%. Mortality was higher in elderly patients, those with a high ASA class, pertrochanteric fracture and males.

**Conclusions:** The present study showed that NA is by far the most common anaesthetic technique for acute hip fracture surgery in Sweden. However, the anaesthetic technique used during this type of surgery had no impact on the 30-day mortality rate in patients. Increasing age, ASA class and male gender increased the 30-day mortality.

## Introduction

Hip fractures affect worldwide 1.6 million patients yearly and the incidence is rising, often elderly patients with comorbidities ^1^. There are annually approximately 17,500 patients with hip fracture in Sweden, the majority being females and elderly https://rikshoft.se/wp-content/uploads/2013/07/rikshoft_rapport2016.pdf. The search for safe and effective anaesthetic techniques for the management of the elderly patient with fracture is still on-going. There are several techniques possible, all with various benefits and potential negative effects. Neuraxial techniques (spinal and epidural) have the benefit of avoiding the need for airway management and only minor effects on cerebral function. However, blood pressure may drop, which is associated with spinal bupivacaine, and there are data showing a drop in blood pressure being a major risk factor ^2^. Neuraxial anaesthesia and oral anticoagulants is also a matter of discussion ^3^. Delay surgery to await the anticoagulant elimination may not be optimal ^4^. The most recent meta-analysis has not been able to show any clear benefit comparing neuraxial and general anaesthesia ^5, 6^.

The aim of the present study was to assess the choice of main anaesthetic technique for acute hip fracture surgery in patients ≥50 years old and the impact of main anaesthetic technique on the 30-day mortality in Sweden. The primary outcome was the impact of anaesthetic technique, general vs. neuraxial, on 30-day mortality. Secondary outcomes were effects of age, sex, ASA class, fracture type and surgery within and after 24 hours on the 30-day mortality.

## Methods

This was a retrospective register study. Ethical permission for the study was obtained from The Regional Ethical Review Board in Stockholm (Dnr: 2017/1915-31; approved 2017-11-08, Annika Sandström). Patient informed consent is not required for register studies in accordance with Swedish research regulations.

The Swedish Perioperative Register (SPOR) data for January 1 ^st^ 2016 and December 31 ^st^ 2017 was reviewed. A diagnosis of acute hip fracture (fracture on the femur as collum fracture (S72.0), pertrochanteric fracture (S72.10) and subtrochanteric fracture (S72.2)), age above 50 years, emergent surgery (within 14 days) and information about 30-day mortality was inclusion criteria for analysis.

The data-sheets retrieved from SPOR for the study analysis were based on the above inclusion criteria and SPOR had helped to categorise anaesthesia into three groups: neuraxial anaesthesia with and without sedation (NA); general anaesthesia (GA); and combined general and neuraxial anaesthesia (CA).

### Statistics

All data is presented as mean and standard deviation. Category data is presented as frequencies and presented as numbers and percent. Difference in mortality was studied by Chi-square test. Continuous variables were analysed by ANOVA and Student-t-test. A p-value < 0.05 was considered statistically significant. Odds ratio and confidence intervals non-adjusted and adjusted were calculated for the primary study variable and the main confounding factors. This is a retrospective register study; thus, no power analysis has been conducted. All statistical analyses were performed using IBM ^®^ SPSS Statistics ^®^ for Macintosh version 24 (Armonk, New York, USA) and Microsoft Excel © 2017 version 16.9.

## Results

**Figure 1.**
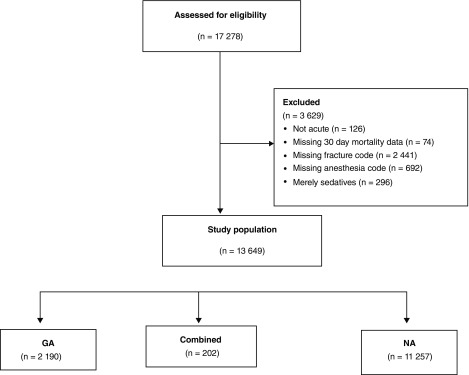
Study flow diagram displaying exclusions for the study cohort.

A total of 13,649 patients were included in the analysis ( Figure 1); 4,601 males and 9,048 females with a mean age of 82 ± 9.6 years. Patients’ demographics are presented in Table 1.

**Table 1.** Primary outcome measures and patient characteristics for GA, NA and CA. Age is presented as mean years (SD). Other results are presented as number of patients (percentage of column). P-value with 95% CI.

	*All* n = 13,649	GA n = 2,190	CA n = 202	NA n = 11,257	*p-value*
**Deceased**	1,050 (7.7)	171 (7.8)	15 (7.4)	864 (7.7)	0.967
***Age*** **50–64** **65–74** **75–84** **85**	81.7 ± 9.6 785 (6) 2,176 (16) 4,539 (33) 6,149 (45)	80.3 ± 9.8 169 (8) 373 (17) 776 (35) 872 (40)	79.4 ± 11.2 25 (12) 37 (18) 57 (28) 83 (41)	82 ± 9.5 591(5) 1,766 (16) 3,706 (33) 5,194(46)	
***Sex*** **Female** **Male**	9,048 (66) 4,601 (34)	1,397 (64) 793 (36)	116 (57) 86 (43)	7,535 (67) 3,722 (33)	
***Fracture*** **Col.** **Per.** **Sub.**	7,365 (54) 5,263 (39) 1,021 (8)	1,233 (56) 784 (36) 173 (8)	100 (50) 81 (40) 21 (10)	6,032 (54) 4,398 (39) 827 (7)	
***ASAPS*** ** 1** ** 2** ** 3** ** 4** ** 5** **Unknown**	525 (4) 4,489 (35) 6,933 (54) 988 (8) 15 (0) 699	30 (2) 519 (25) 1,293 (63) 210 (10) 3 (0) 135	10 (5) 77 (39) 100 (51) 10 (5) 1 (1) 4	485 (5) 3,893 (36) 5,540 (52) 768 (7) 11 (0) 560	
**Surgery** **Within 24 h** **Over 24 h** **Unknown**	13,108 (96) 504 (4) 37	2078 (95) 98 (5) 14	191 (95) 11 () 0	10,839 (96) 395 (4) 23	

Abbreviations: GA = general anesthesia, CA = combined general plus neuraxial anesthesia, NA = neuraxial anesthesia, deceased = 30-day mortality, col = collum femoris fracture, per = pertrochanteric fracture, sub = subtrochanteric fracture, unknown = missing data on variable, ASAPS = American society of Anesthesiologists physical status.

NA (spinal, epidural and combined spinal/epidural) was the most common anaesthetic technique used (82.5% of patients), GA was used in 16% and CA in 1.5% of patients. Mean age was similar between the anaesthetic techniques studied, the proportion of age class 75–84 years and >85 years was however higher among NA compared to GA (79 *vs* 75%; p<0.0001). Sex was evenly distributed: 64 and 67% of GA and NA were female patients, respectively. Collum type fracture was the dominating fracture 56 and 54% of GA and NA patients, respectively. ASA class 3 was the most common functional class with more than 50% of all patients. The proportion of ASA classes 3–5 was higher among GA compared to NA (73 *vs* 59%; p<0.001).

The 30-day mortality for the entire study cohort was 7.7%, with no significant difference between the three anaesthetic techniques studied (GA 7.8%, CA 7.4% and NA 7.7%; Table 1).

Most patients had surgery within 24 hours and there was no difference in delay to surgery between anaesthetic techniques ( Table 2). Duration of anaesthesia, surgery or PACU stay was similar for GA and NA, but somewhat longer CA. There was no clear difference in registered blood loss except for the CA group of patients ( Table 2).

**Table 2.** Outcome measures for subgroup analyzes. Time to surgery, anesthesia time, surgery time and PACU time are presented as means in hours:minutes. Blood loss are presented as means in milliliters.

	*All* n = 13,649	GA n = 2,190	CA n = 202	NA n = 11,257
**Time to surgery**	18:29	20:02	20:01	18:09
**Anesthesia time**	2:10	2:17	2:49	2:08
**Surgery time**	1:09	1:09	1:30	1:09
**PACU time**	4:17	4:17	4:33	4:16
**Blood loss (ml)**	186	200	250	182

Abbreviations: PACU = post anesthesia care unit, GA = general anesthesia, CA = combined general plus neuraxial anesthesia, NA = neuraxial anesthesia

The 30-day mortality was higher among males (10.6%) compared to females (6.2%) and increased for each age class; from 2% among 50–64 years old patients to 11.6% in patients above 85 years of age (see Table 3). There was also significant difference in 30-day mortality between fracture type and with increasing ASA class ( Table 3). The odds ratio for mortality in relation to anaesthetic technique did not change when adjusted for age, sex, type of fracture and ASA class ( Table 4). There was no difference in 30-day mortality between patients that had surgery within 24-hours or later; however the number of patients having surgery beyond 24-hours was small ( Table 5). No differences were seen in duration of anaesthesia, surgery or PACU stay between patients that died compared to survived at day-30 ( Table 5).

**Table 3.** Baseline characteristics and outcome measures for patients undergoing acute hip fracture surgery. Age is presented in years as mean (SD). All other results are presented as number of patients (percentage of column). P-value with 95% CI.

	*All* (n = 13,649)	Deceased (n = 1,050)	Survivors (n = 12,599)	p-value
***Age*** **50–64** **65–74** **75–84** **85**	81.7 (9.6) 785 (6) 2,176 (16) 4,539 (33) 6,149 (45)	86.7 (7.8) 16 (2) 66 (6) 255 (24) 713 (68)	81.3 (9.6) 769 (6) 2,110 (17) 4,284 (34) 5,436 (43)	<0.001
***Sex*** **Female** **Male**	9,048 (66) 4,601 (34)	563 (54) 487 (46)	8,485 (67) 4,114 (33)	<0.001
***Fracture type*** **Collum** **Pertrochanteric** **Subtrochanteric**	7,365 (54) 5,263 (39) 1,021 (8)	521 (50) 444 (42) 85 (8)	6,844 (54) 4,819 (38) 936 (7)	<0.001
***ASAPS*** **1** **2** **3** **4** **5** **Unknown**	525 (4) 4,489 (35) 6,933 (54) 988 (8) 15 (0) 699	3 (0) 124 (13) 639 (64) 219 (22) 8 (1) 57	522 (4) 4,365 (37) 6,294 (53) 769 (6) 7 (0) 642	<0.001
**Surgery** **Within 24 h** **Above 24 h** **Unknown**	13,108 (96) 504 (4) 37	1,011 (96) 38 (4) 1	12,097 (96) 466 (4) 36	0.886

Abbreviations: GA = general anesthesia, CA = combined general plus neuraxial anesthesia, NA = neuraxial anesthesia, deceased = 30-day mortality, Collum = collum femoris fracture, unknown = missing data on variable, ASAPS = American society of Anesthesiologists physical status.

**Table 4.** Odds ratios and confidence intervals for survival unadjusted and adjusted for age, sex, type of fracture and ASA class. Combined anaesthesia, age class 50–64, female collum fracture and ASA 1 was set as reference.

	Deceased Unadjusted odds ratio (CI) (n = 1,050)	Survivors Adjusted odds ratio (CI) (n = 12,599)
**CA** **GA** **NA**	- 0.95 (0.54-1.64) 0.97 (0.56-1.64)	- 0.98 (0.55-1.74) 1.14 (0.94-1.37)
***Age*** **50–64** **65–74** **75–84** **85**	- 0.67 (0.38-1.16) 0.35 (0.21-0.58) 0.16 (0.09-0.27)	- 0.83 (0.46-1.49) 0.48 (0.28-0.82) 0.23 (0.13-0.39)
***Sex*** **Female** **Male**	- 0.56 (0.49-0.64)	- 0.58 (0,50-0,67)
***Fracture type*** **Collum** **Pertrochanteric** **Subtrochanteric**	- 0.83 (0.72-0.95) 0.84 (0.66-1.07)	- 0.88 (0.76-1.02) 0.84 (0.65-1.09)
***ASAPS*** **1** **2** **3** **4** **5**	- 0.20 (0.06-0.64) 0.06 (0.01-0.18) 0.02 (0.00-0.07) 0.01 (0.00-0.03)	- 0.28 (0.08-0.90) 0.1 (0.03-0.31) 0.04 (0.01-0.12) 0.01 (0.00-0.05)

Abbreviations: GA = general anesthesia, CA = combined general plus neuraxial anesthesia, NA = neuraxial anesthesia, deceased = 30-day mortality, Collum = collum femoris fracture, unknown = missing data on variable, ASAPS = American society of Anesthesiologists physical status.

**Table 5.** Number of patients and means for secondary outcomes. Perioperative times are calculated as means and presented as hours:minutes. Blood loss is calculated as means and presented as milliliters. P-value with 95% CI.

	*All* (n = 13,649)	Deceased (n = 1,050)	Survivors (n = 12,599)
**Time to surgery**	18:29	20:01	18:21
**Anesthesia time**	2:10	2:10	2:10
**Surgery time**	1:09	1:06	1:09
**PACU time**	4:17	5:05	4:13
**Blood loss (ml)**	186	180	186

Abbreviations: PACU = post anesthesia care unit, h = hours.

## Discussion

We found NA being by far the most common anaesthetic technique used for acute hip surgery in patients above 50 years of age. However, anaesthetic technique did not impact the 30-day mortality in this retrospective register study in patients having surgery for acute hip fracture. The 30-day mortality increased with age and ASA class. The 30-day mortality was higher in males as compared to females and fracture type also impacted mortality (pertrochanteric fracture was associated to higher mortality).

Our results are in line with previous studies suggesting that anaesthetic technique *per se* does not have a major impact on mortality ^2, 3^. Our overall mortality is also in line with the mortality described in a recent study from the US, including 107,317 hip fracture patients. That study found a 30-day mortality of 8.5% ^7^. Our mortality rate is however somewhat higher than that described by Neuman *et al.* in study published in 2014 from New York ^8^. This study was likewise unable to show any difference in 30-day mortality between general and regional anaesthesia. They did however find a 0.6 day shortened hospital stay in the spinal/epidural group of patients.

There are several limitations of this study. This is a retrospective register study, data derived from the relatively new Swedish perioperative register, SPOR-register ^9^. Registers are dependent on input and data-management, and we are aware that a number of patients were excluded in the analysis of outcome due to missing information. It should also be acknowledged that there are numerous potential alternative anaesthetic techniques for hip fracture surgery. We merely sorted into three main techniques: neuraxial, general and combined anaesthesia. Peripheral blocks and light anaesthesia/sedation may indeed be an option ^10, 11^. We have not considered this surgical technique in the present study.

The focus of our study was to assess the impact of anaesthetic technique on mortality among elderly, patient 50 years and older. Different age limits have been used. We limited our analysis to 50 years and older as pathophysiology reasonably is different; both fracture type/cause and patients’ general health/fragility. The groups were reasonably matched by means of age, fracture type and ASA score.There are without doubt also huge differences in the surgical trauma between merely a screw fixation and a joint prosthesis. We did not explicitly study the impact on anticoagulation, or patients having anticoagulation therapy. A recent paper from the US did not find major differences in complications or death when comparing cohorts of patient without and with anticoagulation therapy; patients having anticoagulation therapy more commonly received GA (84 *vs* 62%) ^12^. We did not sub-group the material further, e.g. cause of fracture and type of surgery. All patients with hip fractures having surgery regardless of cause was included. Combined technique was associated with longer perioperative times and more blood loss than GA and NA. This may be an effect due to combined spinal and epidural anaesthesia being chosen for more complex procedures; however, this is merely speculation. Tight haemodynamic control maintaining blood pressure and heart rate within minimal deviation from preoperative values have been suggested to have a major impact, and studies assessing its effect are being conducted ^13^. Optimising haemodynamics by ultrasound monitoring may also facilitate the perioperative course ^14^. Temperature control is also of importance ^15^. We cannot comment on the anaesthetic protocol performed in the patients included in this study or be more explicit about what drugs were used, nor the handling of any deviation in vital signs. The available register-data does unfortunately not contain information on quality of postoperative care, the occurrence of delirium, postoperative pain and nausea in sufficient fashion for analysis. The postoperative course, mobilisation, ambulation, intake of food and drink and discharge from hospital should indeed be assessed in future studies. Active rehabilitation and physiotherapy is of huge importance ^16^.

Age, comorbidities and increased ASA class are known risk factors for complications after hip fracture surgery ^17, 18^. Nutritional status, malnourishment, as well as obesity, may also have an effect in increasing risk for complications ^19^. We did not use the Nottingham score, and there is without doubt several patient factors that may have contributed to outcome. The International Fragility Fracture Network has recently provided extensive guidelines based on consensus ^20^. Still further studies are indeed warranted to improve the understanding on how to best care for elderly patients with acute hip fracture.

## Conclusion

The study identifies that the majority of hip fracture surgery is performed under spinal anaesthesia in Sweden. Only around 16% of patients undergo general anaesthesia. This could introduce bias. However, the groups appear well matched and the sample size remains large enough for meaningful comparisons. Propensity matching would have increased the fidelity of comparisons but was not performed. The research adds to the current literature identifying that anaesthetic technique has minor impact on 30-day mortality.

Further studies are warranted determining the anaesthetic impact on morbidity, quality of recovery and mortality following high risk orthopaedic surgery.

## Data availability

The data referenced by this article are under copyright with the following copyright statement: Copyright: © 2018 Gremillet C and Jakobsson JG

Data associated with the article are available under the terms of the Creative Commons Zero "No rights reserved" data waiver (CC0 1.0 Public domain dedication). 

The data has been retrieved from the Swedish Perioperative Register (SPOR). This is a national database supported by The National Board of Health and Welfare, Swedish Society for Anaesthesia & Intensive Care and Swedish Association of Local Authorities and Regions and data is thus protected. The data can be retrieved by request from SPOR ( http://www.spor.se/) following Ethical Review board approval via application ( https://www.epn.se/en/start/).
