# Supplementary material for: Acute hip fracture surgery anaesthetic technique and 30-day mortality in Sweden 2016 and 2017: A retrospective register study
Source: F1000Res. 2018 Aug 15;7:1009. Originally published 2018 Jul 5. [Version 2] doi: 10.12688/f1000research.15363.2 (PMC6107981; doi:10.12688/f1000research.15363.2)
Supplement: Peer review history

# Peer review: Referee response for version 2

**DOI:** 10.5256/f1000research.17401.r37214

**Published:** 2018-08-22

**Authors:** Bengt Nellgård

**Affiliations:** 1 Department of Anaesthesiology andIntensive Care Medicine, Sahlgrenska University Hospital, Sahlgrenska Academy, University of Gothenburg, Gothenburg, Sweden

The manuscript by Gremillet and Jaocobsson is now ok.

 I have a minor comment. In discussion line 18 the *i.e*. last sentence on page 4 the say "surgical technique" it should be "anesthetic technique"

 Except for this I have no further comments

I have read this submission. I believe that I have an appropriate level of expertise to confirm that it is of an acceptable scientific standard.

# Peer review: Referee response for version 1

**DOI:** 10.5256/f1000research.16742.r36429

**Published:** 2018-08-02

**Authors:** Colin F. Royse

**Affiliations:** 1 Ultrasound Education Group, Department of Surgery, The University of Melbourne, Melbourne, Vic, Australia

This review examines a specific question of whether general or neuraxial anaesthesia for fractured hip surgery affects 30 day mortality. The paper is well written with appropriate methods and analysis.

 The deficiency of the paper is that it only addresses 30-day mortality and not any metric of quality of survival and quality of recovery. However, these variables may not be available in the SPOR.

1. The data source is not open, but can be obtained with permission from the Swedish Perioperative Registry.
2. Add to the narrative review

The study identifies that the majority of fractured hip surgery is performed under spinal anaesthesia in Sweden. Only around 20% of patients undergo general anaesthesia. This could introduce bias. However, the groups appear well matched and the sample size remains large enough for meaningful comparisons. Propensity matching would have increased the fidelity of comparisons but was not performed. The research adds to the current literature identifying that there is no difference in mortality according to the type of anaesthetic administered.

I have read this submission. I believe that I have an appropriate level of expertise to confirm that it is of an acceptable scientific standard.

# Peer review: Referee response for version 1

**DOI:** 10.5256/f1000research.16742.r36430

**Published:** 2018-07-26

**Authors:** Bengt Nellgård

**Affiliations:** 1 Department of Anaesthesiology andIntensive Care Medicine, Sahlgrenska University Hospital, Sahlgrenska Academy, University of Gothenburg, Gothenburg, Sweden

First of all it is interesting that the authors use the newly started SPOR registry.

 1. The paper needs language improval. I have at least 50 changes in abstract, introduction methods  and results sections. Write in past sense etc Use neutral Words when describing results

 2. Patients less than 65 years are normally not included in studies on hip fracture as they are normally a different entity. Trauma and pathological fractures!!! Are they included? More statistics; GA group is; 2190; C is 202 and; NA is 11247. Can they really get results when comparing the Groups ?

 3. Have they excluded pathological fracture? Reoperations?

 4.In results and figures p values are not clear!!! Does pertrochanteric fractures have higher motrality rate?

 5 Do they have any results on cemented prothesis in cervical fractures? Mortality rate.

 6 Time to surgery; Cut off at 24 h. What do they know about delay 24-36h which is considered ok in  f.ex. UK?

 7 ASA is a crude preoperastive scale, not capturing low hemoglobin, dementia, malignancy and living conditions. Nottingham hip fracture score captures these please comment

 8. Discussion; are there any previous reports from Scandinavia or Sweden addressing the topic? The routine in Sweden seems to be neuraxial anesthesia. This is not the case f.ex. in the USA. Discuss differences?

I have read this submission. I believe that I have an appropriate level of expertise to confirm that it is of an acceptable scientific standard, however I have significant reservations, as outlined above.

# Response

**Published:** 2018-08-01

**Authors:** Jan Jakobsson

**Affiliations:** Department of Physiology and Pharmacology, Karolinska Institutet, Sweden

Responses from the authors;

Dear Referee,

Thank you for valuable comments.

First of all, it is interesting that the authors use the newly started SPOR registry.

1. The paper needs language improval. I have at least 50 changes in abstract, introduction methods  and results sections. Write in past sense etc Use neutral Words when describing results

***Response;*** We will revise language and use past sense and neutral wordings of results.

2. Patients less than 65 years are normally not included in studies on hip fracture as they are normally a different entity. Trauma and pathological fractures!!! Are they included? More statistics; GA group is; 2190; C is 202 and; NA is 11247. Can they really get results when comparing the Groups ?

***Response;*** The focus of our study was to assess the impact of anaesthetic technique on mortality among elderly, patient 65 and older. Different age limits have been used. We limited our analysis to the 65 and 65+ age as pathophysiology reasonably is different; both fracture type/cause and patients’ general health/fragility. The traditional Chi-2-tests should compensate for different in group size and we do believe that bust unadjusted and adjusted results are statistically sound.

3. Have they excluded pathological fracture? Reoperations?

***Response;*** No: All hip fracture undergoing surgery with general, or neuraxial anaesthesia aged 65 and 65+ are included regardless of cause; we have not subgroup patients on trauma energy or bone density or similar.

4.In results and figures p values are not clear!!! Does pertrochanteric fractures have higher mortality rate?

***Response;*** The mortality did differ within each fracture cohort, it was highest among pertrochanteric (8.4%) and lowest among the collum fracture patients (7.1%) We are not able to comment on cause of death, or whether the pertrochanteric patients had more extensive surgery.

5 Do they have any results on cemented prothesis in cervical fractures? Mortality rate.

***Response;*** No we have no data related to surgical technique e.g. use of cement.

6 Time to surgery; Cut off at 24 h. What do they know about delay 24-36h which is considered ok in  f.ex. UK?

***Response;*** As opposed to the findings in this study, some have found a significantly higher risk of 30-day mortality for surgery later than 24 hours (41, 42). Some studies even suggest an increased mortality when surgical delay is more than 12 hours (38, 39) while other suggest 48 hours (36, 43). In a study adjusting for potential confounders, no difference in mortality was found in patients receiving surgery within 3 days as compared to those above 3 days (44). Swedish guidelines advise surgery within 36 to 48 hours and suggest adequate care of the patient and competent staff as equally crucial, although surgery within 24 hours is recommended (5). The results of this study should be interpreted with caution considering only 4% of the patients waited more than 24 hours for surgery. There are differences in the studies potentially explaining difference in result, such as variation in characteristics and size of study population, country and time-period for collection of data and outcome definitions.

7 ASA is a crude preoperastive scale, not capturing low hemoglobin, dementia, malignancy and living conditions. Nottingham hip fracture score captures these please comment,

***Response;*** Most valid comment, we did not use the Nottingham score, and there is without doubt several patient factors that may have contributed to outcome.

8. Discussion; are there any previous reports from Scandinavia or Sweden addressing the topic? The routine in Sweden seems to be neuraxial anesthesia. This is not the case f.ex. in the USA. Discuss differences?

***Response;*** The aim of the study was to use the PSOR register to assess what anaesthetic techniques that are used and whether we from retrospective data could see any difference in 30-day mortality between anaesthetic techniques used. We are not aware of any previous Swedish study assessing anaesthetic techniques independent impact on 30-day mortality.
